# A stochastic model of myeloid cell lineages in hematopoiesis and pathway mutations in acute myeloid leukemia

**DOI:** 10.1371/journal.pone.0204393

**Published:** 2018-10-01

**Authors:** Frank Jäkel, Oliver Worm, Sascha Lange, Roland Mertelsmann

**Affiliations:** 1 Psiori GmbH, Freiburg, Germany; 2 Innere Medizin I, Universitätsklinikum Freiburg, Freiburg, Germany; Beijing Key Laboratory of Diabetes Prevention and Research, CHINA

## Abstract

A model for hematopoiesis is presented that explicitly includes the erythrocyte, granulocyte, and thrombocyte lineages and their common precursors. A small number of stem cells proliferate and differentiate through different compartments to produce the vast number of blood cells needed every day. Growth factors regulate the proliferation of cells dependent on the current demand. We provide a steady state analysis of the model and rough parameter estimates. Furthermore, we extend the model to include mutations that alter the replicative capacity of cells and introduce differentiation blocks. With these mutations the model develops signs of acute myeloid leukemia.

## Introduction

All blood cells are generated from very few stem cells and go through several stages of cell division and differentiation that greatly amplify the number of cells. In fact, one cell division per day at the stem cell stage is thought to lead to roughly 350 billion cells flowing out into the blood stream every day. How is this massive amplification achieved? And how does this process explain the dynamical changes in blood cell counts that clinicians observe in their daily work, e.g. in leukemia?

There is a long history of mathematical modeling of hematopoiesis with two traditions, one rooted in differential equations and one in stochastic modeling [1, 2]. The dynamical and control-theoretic aspects of hematopoiesis are naturally captured with differential equations. In contrast, the detailed biology of cell proliferation and differentiation is often easier to model with discrete stochastic processes, which often go down to the single cell level and sometimes even include genetic and other intracellular processes. This tension between single cell models and models of the global dynamics is in no way unique to hematopoiesis, it exists in all areas of systems biology.

However, a specific challenge in hematopoietic modeling is that the whole system crucially depends on a very small number of hematopoietic stem cells, making it very desirable to have models that span the micro- and the macro-level [3]. Also, biomedical research on the pathologies of the hematopoietic system increasingly focuses on molecular and genetic explanations. For example, the genes that are associated with human myeloid leukemia are extremely well characterized [4–7] and cancerogenesis, in general, is now understood as arising from a very small number of mutations in a variety of pathways that tightly regulate cell proliferation and cell death [8–10]. These genetic and molecular insights can be incorporated into models of the global dynamics [11, 12] but without modeling single cells the effects of single mutations on leukemogenesis cannot be studied directly.

Here, we present a stochastic, compartmental model that counts single cells at various stages of hematopoiesis. Our model is strongly inspired by the model of Dingli et al. [13] that was later generalized and analyzed in detail by Werner et al. [14]. In the original model no distinction between different cell types is made and hence the different characteristics of, for example, the erythrocyte, granulocyte, and thrombocyte lineages in hematopoiesis cannot be taken into account. The major extension we propose here is to explicitly model these three myeloid lineages of hematopoiesis. In addition, we will also include a feedback mechanism with lineage-specific growth factors. As we account for the three lineages and their common precursors the feedback mechanisms that we propose is much more detailed than previous extensions of the original model that also included feedback [15]. Furthermore, setting the parameters of our model to realistic values is harder than in the original model because of interactions between the three lineages. We show, however, that rough parameter estimates can still be obtained by considering the steady state, similar to how Dingli et al. [13] did it. Finally, we extend the model to include single mutations that might account for some aspects of acute myeloid leukemia (AML). In this regard, our model mirrors similar efforts by Werner and colleagues [14, 16, 17], who do not, however, deal with the complications of differentiating between cell lineages.

## Methods

Even though our model is based on the model of Dingli et al. [13], the introduction of different cell lineages and the inclusion of cell-lineage specific growth factors make it easier to explain our model from scratch, rather than to present it as an extension of the original model. This is what we will do in the *Methods* section. The *Results* section will then give a theoretical analysis of the new model and show that based on this analysis the model’s parameters can be set to physiologically plausible values. Finally, we will extend the model slightly to allow for single mutations in single cells and use this extension to simulate the development of acute myeloid leukemia.

### A compartmental model

We will consider the numbers of three myeloid types of blood cells: erythrocytes (*E*), granulocytes (*E*), and thrombocytes (*T*). To keep the model simple, we ignore the monocytes and only consider the granulocytes among the leukocytes. Risk of bacterial infections among transplant patients is predominantly correlated with the number of neutrophil leukocytes and therefore the granulocyte count is clinically the more relevant variable. Also, the monocytes only make up a very small proportion of the leukocytes anyway. In addition we will consider the common precursors (*C*) of the myeloid cells, including the hematopoietic stem cells.

**Fig 1:**
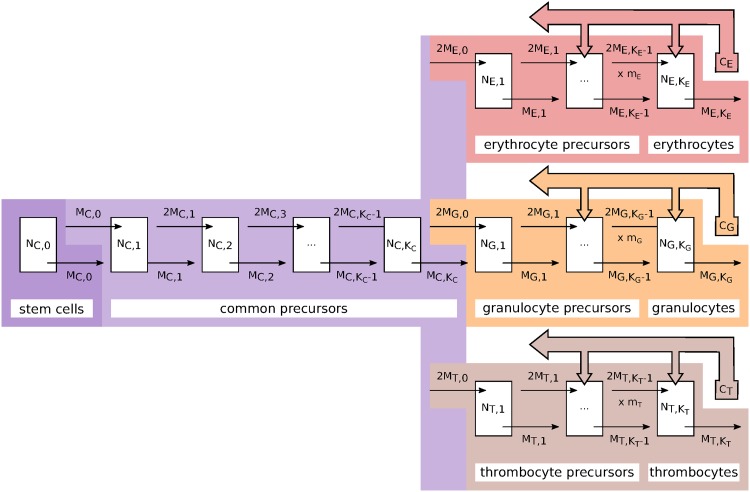
Illustration of the model’s compartments. The model consists of *K*_*C*_ + 1 compartments for the common precursors. The zeroth compartment is the stem cell compartment. The erythrocyte lineage has *K*_*E*_ compartments, and the granulocyte and thrombocyte lineages have *K*_*G*_ and *K*_*T*_ compartments, respectively. The number of cells in each compartment is given by *N* and the outflow of each compartment by *M* with the respective indices. The cells at the final stage (on the very right) model the mature blood cells in the blood stream. Deviations of the respective blood counts from their normal range result in lineage specific growth factors (*c*_*E*_, *c*_*G*_, *c*_*T*_) that feed back to all stages and increase their outflow.

We assume there are *K*_*E*_, *K*_*G*_ and *K*_*T*_ compartments for the erythrocyte, granulocyte, and thrombocyte lineages. For the common precursors we assume there are *K*_*C*_ + 1 compartments with the zeroth compartment being the stem cell compartment. Each compartment has *N*_*x*,*k*_(*t*) cells in it at time point *t*. For example, we assume the zeroth compartment of the common precursors is the stem cell compartment and *N*_*C*,0_(*t*) is the number of cells in it. $N_{C,K_{C}}\left(t\right)$ is the last stage before cells commit themselves to one of the three lineages. *N*_*E*,1_(*t*) is then the first stage of the erythrocyte lineage and $N_{E,K_{E}}\left(t\right)$ is the number of erythrocytes in the blood. Likewise for the granulocytes and the thrombocytes. Fig 1 illustrates the compartments of the model. Furthermore, there are a number of growth factors in the bone marrow that change over time. We collect the (log) concentrations for all these growth factors in *c*(*t*) = {*c*_*E*_(*t*), *c*_*G*_(*t*), *c*_*T*_(*t*)}. These growth factors act as a feedback signal to control the outflow of each stage to ensure that the blood counts in the blood stream maintain their normal levels. The rate at which cells leave their compartment therefore depends on these concentrations: *r*_*x*,*k*_(*c*(*t*)) measured in 1/days for *x* ∈ {*C*, *E*, *G*, *T*}.

To simulate the number of cells in each compartment over time we discretize time, t∈{jΔt∣j∈N} measured in days. With Δ*t* sufficiently small the probability that a cell divides itself in any one time step is

$$\begin{array}{c} r_{x,k}^{\prime }\left(t\right)=r_{x,k}\left(c\left(t\right)\right)\Delta t. \end{array}$$ (1)

For Δ*t* → 0 this discrete process becomes an inhomogeneous Poisson process. Since we have *N*_*x*,*k*_(*t*) cells in the *k*th compartment, the number *M*_*x*,*k*_(*t*) of the cells that leave this compartment at time *t* is a Binomial distribution with

$$\begin{array}{c} M_{x,k}\left(t\right)∼\text{Binomial}\left(N_{x,k}\left(t\right),r_{x,k}^{\prime }\left(t\right)\right) \end{array}$$ (2)

for *x* ∈ {*C*, *E*, *G*, *T*} and all the relevant *k*.

#### Stem cell compartment

Cell division in the stem cell compartment is asymmetrical: If a cell divides, it turns into two daughter cells, one daughter cell is a clone of the stem cell and stays in the stem cell compartment and the other cell differentiates and leaves the stem cell compartment. Asymmetric cell division assures that the number of cells in the stem cell compartment, *N*_*S*_, stays constant as new cells leave it, hence

$$\begin{array}{c} N_{C,0}\left(t+\Delta t\right)=N_{C,0}\left(t\right)=N_{S} \end{array}$$ (3)

for all *t*. *M*_*C*,0_(*t*) cells leave the stem cell compartment at each time step and enter the next compartment, i.e. the first compartment of the common precursors.

#### Common precursor compartments

The next compartment’s size over time is

$$\begin{array}{c} N_{C,1}\left(t+\Delta t\right)=N_{C,1}\left(t\right)-M_{C,1}\left(t\right)+M_{C,0}\left(t\right), \end{array}$$ (4)

where *M*_*C*,1_(*t*) is the number of cells that leave it. The cells that leave this compartment divide symmetrically: Cells that divide themselves turn into two daughter cells and each of them differentiates and flows on to the next compartment. Contrary to previous work [13] we do not take into account potential self-renewal. With this simplification the inflow is 2*M*_*C*,*k*−1_ and

$$\begin{array}{c} N_{C,k}\left(t+\Delta t\right)=N_{C,k}\left(t\right)-M_{C,k}\left(t\right)+2M_{C,k-1}\left(t\right) \end{array}$$ (5)

for all subsequent compartments 1 < *k* ≤ *K*_*C*_ (see Fig 1). As there is no self-renewal in any of the non-stem-cell compartments the number of cell divisions a cell has gone through since it left the stem-cell compartment automatically determines its stage of maturation.

#### Committed precursor compartments and blood stream

What is the number of cells in the first compartment of each lineage? We assume that cells at the last common precursor stage randomly decide to go into one of the three blood cell lineages with probabilities *q*_*E*_(*c*(*t*)), *q*_*G*_(*c*(*t*)), and *q*_*T*_(*c*(*t*)) that depend on the current growth factor concentrations *c*(*t*):

$$\begin{array}{c} \left(\begin{array}{c} M_{E,0}\left(t\right)\\ M_{G,0}\left(t\right)\\ M_{T,0}\left(t\right) \end{array})∼\text{Multinomial}(M_{C,K_{C}}\left(t\right),(\begin{array}{c} q_{E}\left(c\left(t\right)\right)\\ q_{G}\left(c\left(t\right)\right)\\ q_{T}\left(c\left(t\right)\right) \end{array})\right) \end{array}$$ (6)

for all *t*. Cells that leave the last compartment of the common precursors divide symmetrically, as do all all subsequent compartments, and hence the outflow from the previous stage is always multiplied by two:

$$\begin{array}{c} N_{x,k}\left(t+\Delta t\right)=N_{x,k}\left(t\right)-M_{x,k}\left(t\right)+2M_{E,k-1}\left(t\right) \end{array}$$ (7)

for *x* ∈ {*E*, *G*, *T*} and 0 < *k* < *K*_*x*_. The last stage of each linage models the blood stream, i.e. the number of erythrocytes $N_{E,K_{E}}$, granulocytes $N_{G,K_{G}}$, and thrombocytes $N_{T,K_{T}}$. Thrombocytes in the blood stream are not cells, instead platelets are generated from megakaryocytes and each megakaryocyte nuclear unit gives rise to many platelets. Hence, in order to be able to accommodate this fact we multiply the outflow of the previous to last stage with an additional factor *m*_*x*_, therefore

$$\begin{array}{c} N_{x,K_{x}}\left(t+\Delta t\right)=N_{x,K_{x}}\left(t\right)-M_{x,K_{x}}\left(t\right)+2M_{x,x_{T}-1}\left(t\right)\cdot m_{x} \end{array}$$ (8)

for *x* ∈ {*E*, *G*, *T*}. For the erythrocyte and granulocyte lineages *m*_*E*_ and *m*_*G*_ will be set to 1 whereas for the thrombocyte lineage *m*_*T*_ will be the number of platelets in a megakaryocyte nuclear unit.

#### Proliferation and death rates

It is known that within the bone marrow the proliferation rates increase with further differentiation (i.e. *k*). Stem cells divide only about once a year and the last committed precursors can divide several times a day. Dingli et al. [13] make the assumption that all rates are constant over time and that the ratio of rates *r* = *r*_*x*,*k*_/*r*_*x*,*k*−1_ at subsequent stages is constant, too. Therefore, *r*_*x*,*k*_ is *r*^*k*^*r*_*x*,0_ for 1 < *k* ≤ *K*_*x*_ and proliferation rates increase exponentially with differentiation stage *k*.

Here, we will make a similar but different assumption. The division rates at each stage should be controlled by the concentration of relevant growth factors (e.g. SCF, GM-CSF, Epo, G-CSF, TPO, etc.). We make the simplifying assumption that there are just three *effective* growth factors, one for each lineage. These could be Epo for the erythrocyte, G-CSF for the granulocyte, and TPO for the thrombocyte lineage—but more likely there is a combination of several factors in each lineage.

Each of the growth factors for the three lineages *x* ∈ {*E*, *G*, *T*} has a certain concentration at each time point. Instead of using the concentrations for each growth factor directly it will be more convenient to parameterize the model with the logarithm of the concentrations *c*_*x*_(*t*). Let us again use the shorthand *c*(*t*) = {*c*_*E*_(*t*), *c*_*G*_(*t*), *c*_*T*_(*t*)} for the collection of all three concentrations. The division rates of a cell will depend on the probability that growth factors are bound to its receptors—which in turn depends on the growth factor concentrations. Note that the rates for each cell type can potentially depend on the concentrations of all three growth factors. In fact, we make the assumption that all common precursors (including the stem cells) have receptors for each of the three growth factors and in order to be able to divide, common precursors have to have at least one bound growth factor. In contrast, the committed precursors for the three lineages are only sensitive to their specific growth factor.

Let *s*_*x*,*j*_ be the sensitivity for the specific growth factor of each lineage *x* ∈ {*E*, *G*, *T*} at stage *j* where 0 ≤ *j* < *K*_*C*_ + *K*_*x*_ (i.e. looking at Fig 1 the stem cells have index *j* = 0 and the first compartment of each committed lineage has index *j* = *K*_*C*_ + 1). The indices 0 ≤ *j* ≤ *K*_*C*_ capture the sensitivity of the common precursors to each of the three growth factors. For example, *s*_*E*,0_ is the sensitivity of the stem cells to the growth factor of the erythrocyte lineage and $s_{E,K_{C}}$ is the sensitivity of the last common precursor. The indices *K*_*C*_ < *j* < *K*_*C*_ + *K*_*x*_ capture the sensitivities of the committed precursors. As cells at stage *K*_*C*_ + *K*_*E*_ are the mature erythrocytes in the blood stream, the last stage that is sensitive to the *E* growth factor is *K*_*C*_ + *K*_*E*_ − 1. We assume that *s*_*x*,*j*_ is a simple linear function of *j*, i.e. *s*_*x*,*j*_ = *a*_*x*_ *j* + *b* for 0 ≤ *j* < *K*_*C*_ + *K*_*x*_ with positive slope *a*_*x*_ and constant offset *b*. This is the key restriction in our model that replaces the constant ratio assumption of Dingli et al. [13]. Given these sensitivities the simplest model for the probability that a ligand is bound to a receptor is given by a logistic function:

$$\begin{array}{c} p_{x,j}\left(c\right)=\frac{1}{1+e^{-a_{x}j-b-c_{x}}}\;\text{for}\;0\leq j<K_{C}+K_{x}, \end{array}$$ (9)

where with a slight abuse of notation *c* = *c*(*t*) is the collection of the logarithms of the three ligand concentrations and *c*_*x*_ pulls out the relevant concentration from *c*. If the diffusion of the growth factors in the bone marrow is much faster than the division rate of the cells, the binding of growth factors can be considered to be in steady state relative to the hematopoietic system. Under this assumption the equation for *p*_*x*,*j*_(*c*) can be derived from the principles of statistical mechanics (see e.g. the discussion of Hill functions in [18]).

**Fig 2:**
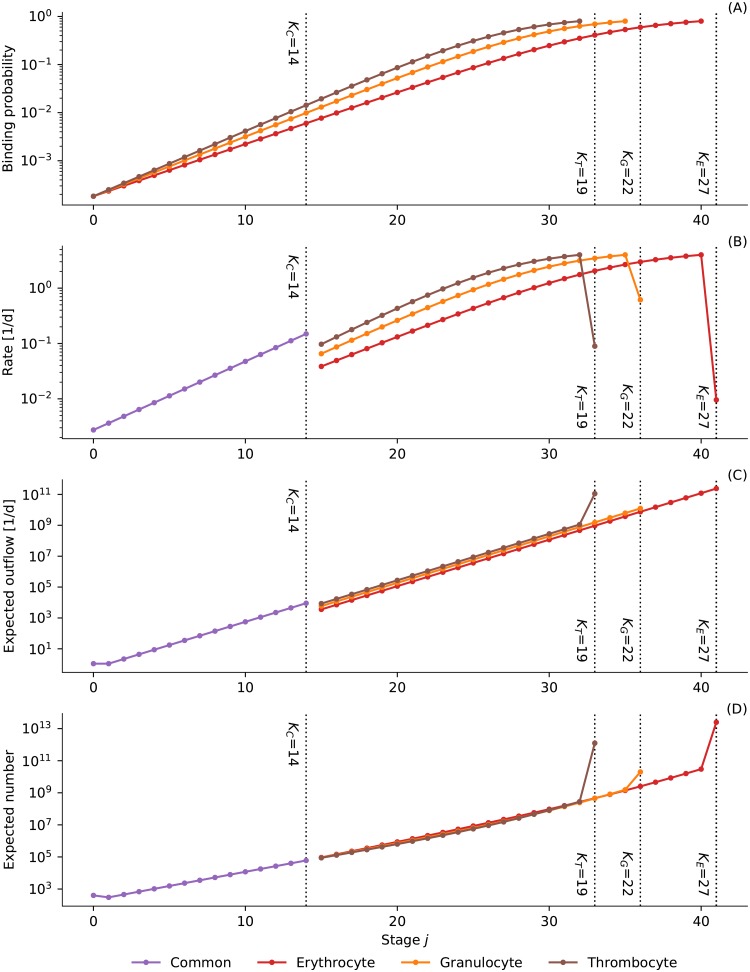
Model stages. Panel (A) shows binding probabilities for the erythrocyte, granulocyte, and thrombocyte growth factors as a function of the differentiation stage. Panel (B) shows the corresponding proliferation rates, except for the last stages of each lineage that model the blood stream. For those the rates correspond to the death rates of erythrocytes, granulocytes, and thrombocytes. Panels (C) and (D) depict the expected outflow and number of cells at each stage. All curves are for the growth factor concentrations at their target values (all *c*_*x*_ = 0) and with all parameters set to realistic values.

Panel (A) in Fig 2 shows the binding probabilities for the three growth factors as a function of the stage *j* for all *c*_*x*_ = 0 (and *a*_*x*_, *b*, *K*_*C*_ and *K*_*x*_ set to realistic values). If the concentrations increase the curves will shift to the left and the binding probabilities increase at all stages. For a decrease in concentrations the curves shift to the right and binding probabilities decrease.

We assume there is a maximum division rate *r*_max_ that is determined by the cell cycle. This maximum rate is the same for all cell types. The division rate of each cell type reduces to the fraction of cells that have growth factors bound. For the common precursors we assume that they have three receptors, one for each growth factor of the respective lineage. We further assume they can divide if any of the three receptors have a growth factor bound to them. The probability that this happens at the common precursor stage *k* is the same as one minus the probability that none of the growth factors are bound and hence the rate at which these cells divide is

$$\begin{array}{c} r_{C,k}\left(c\right)=r_{\text{max}}\cdot \left(1-\left(1-p_{E,k}\left(c\right)\right)\left(1-p_{G,k}\left(c\right)\right)\left(1-p_{T,k}\left(c\right)\right)\right) \end{array}$$ (10)

for 0 ≤ *k* ≤ *K*_*C*_. The committed progenitors of each lineage only have a receptor for their respective growth factor and their proliferation rates are accordingly

$$\begin{array}{c} r_{x,k}\left(c\right)=r_{\text{max}}\cdot p_{x,K_{C}+k}\left(c\right) \end{array}$$ (11)

for *x* ∈ {*E*, *G*, *T*} and 1 ≤ *k* < *K*_*x*_. The last stages *K*_*E*_, *K*_*G*_, and *K*_*T*_ of the three lineages are the erythrocytes, granulocytes, and thrombocytes in the blood stream. The rates at which each of them leaves the blood stream is assumed to be constant and is only determined by their respective death rates, i.e for each *x* ∈ {*E*, *G*, *T*}

$$\begin{array}{c} r_{x,K_{x}}\left(c\right)=r_{x}, \end{array}$$ (12)

and is therefore independent of the growth factor concentrations *c*. Note that the only way for a cell to die is to become a fully mature blood cell and then be cleared out of the blood stream after having served its purpose. Of course, in a more realistic model premature cells should also die sometimes. As it is hard to experimentally estimate the death rates for the various stages we have decided to ignore this possibility.

Panel (B) of Fig 2 gives a concrete numerical example of how the rates change for the four cell types as a function of the stage and again with all *c*_*x*_ = 0 (with *r*_max_ and all *r*_*x*_ set to realistic values). There is a pronounced jump at *K*_*C*_ as the common progenitors have receptors for all three growth factors and the last common progenitor at *K*_*C*_ is therefore more active than each committed progenitor at the next stage. The rates drop to the death rates at the last stage of each lineage as these are not dependent on the binding probabilities (panel A). For the lower stages the rates look linear in log space and hence increase exponentially, as in the model of Dingli et al. [13]. In contrast to their model the rates in our model saturate at *r*_max_.

#### From last common precursor to the three lineages

We will use the binding probabilities from Eq (9) to set the probabilities *q*_*E*_(*c*), *q*_*G*_(*c*) and *g*_*T*_(*c*) that common precursor cells enter the three lineages (see Eq 6). Let us look at the erythrocyte lineage first. We will assume that if a cell at the last stage of the common precursors has only an *E* growth factor bound and not any of the other two then the cell will enter the erythrocyte lineage. If it has an *E* growth factor and exactly one of the other two, the probability to enter the *E* lineage is 1/2. If all growth factors are bound the probability is 1/3. Let

$$\begin{array}{cc} q_{E}^{\prime }\left(c\right) & =p_{E,K_{C}}\left(c\right)\left(1-p_{G,K_{C}}\left(c\right)\right)\left(1-p_{T,K_{C}}\left(c\right)\right)\\ & +\frac{1}{2}p_{E,K_{C}}\left(c\right)p_{G,K_{C}}\left(c\right)\left(1-p_{T,K_{C}}\left(c\right)\right)+\frac{1}{2}p_{E,K_{C}}\left(c\right)\left(1-p_{G,K_{C}}\left(c\right)\right)p_{T,K_{C}}\left(c\right)\\ & +\frac{1}{3}p_{E,K_{C}}\left(c\right)p_{G,K_{C}}\left(c\right)p_{T,K_{C}}\left(c\right) \end{array}$$

and similarly for $q_{G,K_{C}}^{\prime }$ and $q_{T,K_{C}}^{\prime }$. With these three values we can compute the probability that a cell that divides at the last common precursor stage enters lineage *x* ∈ {*E*, *G*, *T*} as

$$\begin{array}{c} q_{x}\left(c\right)=\frac{q_{x}^{\prime }\left(c\right)}{q_{E}^{\prime }\left(c\right)+q_{G}^{\prime }\left(c\right)+q_{T}^{\prime }\left(c\right)}. \end{array}$$ (13)

#### Feedback mechanism

The last missing component for the model is the specification of the feedback mechanism. We assume that feedback regulates the growth factor concentration for each lineage *x* ∈ {*E*, *G*, *T*} separately. If the number $N_{x,K_{x}}\left(t\right)$ of cells or platelets in the blood is lower than the target number *N*_*x*_ the concentration goes up, otherwise it goes down, i.e.

$$\begin{array}{c} c_{x}\left(t\right)=\frac{\delta }{N_{x}}(N_{x}-N_{x,K_{x}}\left(t\right)) \end{array}$$ (14)

where *δ*/*N*_*x*_ is the gain of the feedback mechanism that, for simplicity, we assume to be the same for all three growth factors relative to the respective target number *N*_*x*_. Hence, the feedback mechanism tries to keep all *c*_*x*_(*t*) at zero and therefore $N_{x,K_{x}}\left(t\right)$ close to *N*_*x*_. While the feedback signal is a linear function of the deviation, its effect on the binding probabilities (Eq 9)—and therefore the proliferation rates at each stage (Eq 11)—is non-linear. Also, the feedback mechanism is deterministic and will reduce the overall noise in the system. In fact, we will see below (subsection on *noise scaling*) that the system behaves almost deterministically and extend it in a way that better reflects the noise in biological systems.

## Results

Now that we have presented our model in full detail, the *Results* section will first give a theoretical analysis of the steady-state behavior of the model. Then this analysis will be used to set the model’s parameters to physiologically sensible values. Finally, we extend the model slightly to allow for simulating the effects of single mutations on leukemogenesis.

### Conditional steady state analysis

The proliferation rates at all stages and the probability of cells to enter each lineage depend on the binding probabilities in Eq (9). These, in turn, are completely determined by the growth factor concentrations *c*_*x*_(*t*) in the bone marrow. The concentrations for the three growth factors therefore control the overall output of cells for all three lineages and are themselves controlled by the feedback mechanism that keeps the number of erythrocytes, granulocytes, and thrombocytes close to their target values (Eq 14). To better understand this system we will analyze its steady state behavior for given, fixed concentrations *c*_*x*_(*t*) = *c*_*x*_. For the following derivations we will again collect these three numbers in one variable *c* = {*c*_*E*_, *c*_*G*_, *c*_*T*_}.

In steady state the expected number of cells at each state is constant and the expected inflow equals the expected outflow on all stages. For the common precursors this means that Eq (4) can be simplified to

$$\begin{array}{c} E\left[M_{C,1}|c\right]=E\left[M_{C,0}|c\right] \end{array}$$

where we have dropped the dependence on *t* since all time points behave in the same way in steady state. By the same reasoning Eq (5) can be simplified to

$$E[M_{C,k}|c]=2E[M_{C,k-1}|c]\;\text{for}\;1<k\leq K_{C}.$$

From these two equations it follows that

$$E[M_{C,k}|c]=2^{k-1}E\left[M_{C,0}|c\right]\;\text{for}\;1<k\leq K_{C}.$$

For the three downstream lineages *x* ∈ {*E*, *G*, *T*} the same reasoning can be applied to Eqs (7) and (8) and results in

$$\begin{array}{cc} E[M_{x,k}|c]=2^{k}E\left[M_{x,0}|c\right] & \;\text{for}\;1<k<K_{x}\\ E[M_{x,k}|c]=2^{k}E\left[M_{x,0}|c\right]\cdot m_{x} & \;\text{for}\;k=K_{x} \end{array}$$

where again the last stage of each lineage *K*_*x*_ needs to be treated differently because there is the additional factor *m*_*x*_ to accommodate the fact that megakaryocytes burst into *m*_*T*_ platelets.

The expected inflow for all three lineages *x* ∈ {*E*, *G*, *T*} depends on the expected outflow of the last common progenitor stage and the probability *q*_*x*_(*c*) that a cell enters lineage *x* (Eq 6) is

$$\begin{array}{c} E\left[M_{x,0}|c\right]=E\left[M_{C,K_{C}}|c\right]\cdot q_{x}\left(c\right) \end{array}$$

because *c* is given and therefore *q*_*x*_(*c*) is not random. Since $E\left[M_{C,K_{C}}|c\right]=2^{K_{C}-1}E\left[M_{C,0}|c\right]$ the preceding equations can be summarized as

$$\begin{array}{cc} E[M_{C,k}|c]=E\left[M_{C,0}|c\right]\cdot 2^{k-1} & \;\text{for}\;1\leq k\leq K_{C} \end{array}$$ (15)

$$\begin{array}{cc} E[M_{x,k}|c]=E\left[M_{C,0}|c\right]\cdot 2^{K_{C}+k-1}\cdot q_{x}\left(c\right) & \;\text{for}\;1\leq k<K_{x} \end{array}$$ (16)

$$\begin{array}{cc} E[M_{x,k}|c]=E\left[M_{C,0}|c\right]\cdot 2^{K_{C}+k-1}\cdot q_{x}\left(c\right)\cdot m_{x} & \;\text{for}\;k=K_{x} \end{array}$$ (17)

where *x* ∈ {*E*, *G*, *T*}. This shows that the expected outflow of all stages *only* depends on the expected outflow of the stem cell compartment *E*[*M*_*C*,0_∣*c*]. Hence, using these equations the expected outflow for all compartments can be computed by starting with the expected outflow of the stem cell compartment

$$\begin{array}{c} E\left[M_{C,0}|c\right]=E\left[N_{C,0}|c\right]r_{C,0}\left(c\right)\cdot \Delta t=N_{S}r_{C,0}\left(c\right)\cdot \Delta t \end{array}$$

where we have used that *M*_*C*,0_ is binomially distributed (Eq 2) and that the number of stem cells is constant (Eq 3). Panel (C) in Fig 2 shows the expected outflow of cells per day for each stage for realistic parameter values and all *c*_*x*_ = 0. The constant slope in log space is due to the doubling at each stage for all cell types—except for the stem cell compartment and the last stage of the thrombocyte lineage. In the former case there is asymmetric cell division and therefore the outflow of the stem cell compartment and the next compartment are the same. In the latter case each megakaryocyte results in many more than just two platelets (*m*_*T*_ ≫ 1 whereas *m*_*E*_ = *m*_*G*_ = 1).

From the expected outflow we can compute the expected number of cells at each stage. For all the left hand sides of Eqs (15), (16) and (17)

$$\begin{array}{c} E\left[M_{x,k}|c\right]=E\left[N_{x,k}|c\right]r_{x,k}\left(c\right)\cdot \Delta t \end{array}$$

because, again, the *M*_*x*,*k*_ are binomially distributed (Eq 2). Plugging the last two equations back into Eqs (15), (16) and (17) we find that the expected number of cells at each stage is

$$\begin{array}{cc} E[N_{C,k}|c]=\frac{N_{S}r_{C,0}\left(c\right)}{r_{C,k}\left(c\right)}\cdot 2^{k-1} & \text{for}\;1\leq k\leq K_{C} \end{array}$$ (18)

$$\begin{array}{cc} E[N_{x,k}|c]=\frac{N_{S}r_{C,0}\left(c\right)}{r_{x,k}\left(c\right)}\cdot 2^{K_{C}+k-1}\cdot q_{x}\left(c\right) & \text{for}\;1\leq k<K_{x} \end{array}$$ (19)

$$\begin{array}{cc} E[N_{x,k}|c]=\frac{N_{S}r_{C,0}\left(c\right)}{r_{x,k}\left(c\right)}\cdot 2^{K_{C}+k-1}\cdot q_{x}\left(c\right)\cdot m_{x} & \text{for}\;k=K_{x}. \end{array}$$ (20)

Panel (D) in Fig 2 shows the number of expected cells for each stage in the hematopoietic system for realistic parameter values and all *c*_*x*_ = 0. The expected number drops a little in the first compartment after the stem cell compartment because the rate increases (panel B) but the outflow does not (panel C). The big jumps on entering the blood stream at *K*_*C*_ + *K*_*x*_ are due to the fact that the death rates *r*_*x*_ are much lower than the proliferation rates of the last progenitors (panel B).

The blood stream, i.e. the last stage *K*_*x*_ of each lineage, is also special because it produces the feedback to all the other stages of the hematopoietic system. The concentration of each growth factor *c*_*x*_ is a deterministic and one-to-one function of the number of cells in each last compartment $N_{x,K_{x}}$ (Eq 14). Hence, if *c*_*x*_ is known so is $N_{x,K_{x}}$. In particular, if *c*_*x*_ = 0

$$\begin{array}{c} E\left[N_{x,K_{x}}|c=0\right]=N_{x} \end{array}$$

where we used the abbreviation *c* = 0 to mean all *c*_*x*_ = 0 in *c* = {*c*_*E*_, *c*_*G*_, *c*_*T*_}. Using the same abbreviation let *r*_*S*_ = *r*_*C*,0_(0) be the proliferation rate of the stem cells for *c* = 0, i.e. this is the rate that the feedback mechanism aims for (Eq 14). Remember that $r_{x}=r_{x,K_{x}}\left(0\right)$ are the death rates of each lineage in the blood stream independent of the growth factor concentrations (Eq 12). Plugging all these values into Eq (20), the conditional expected number of erythrocytes, granulocytes, and thrombocytes at the final stages hence have to fulfill the following constraints:

$$\begin{array}{c} N_{x}=\frac{N_{S}r_{S}}{r_{x}}\cdot 2^{K_{C}+K_{x}-1}\cdot q_{x}\cdot m_{x} \end{array}$$ (21)

for *x* ∈ {*E*, *G*, *T*} and with *q*_*x*_ = *q*_*x*_(0).

Furthermore, let $N_{C}=E\left[N_{C,K_{C}}|c=0\right]$ and $r_{C}=r_{C,K_{C}}\left(0\right)$ be shorthands for the respective values of the last common progenitor compartment. Plugging these into Eq (18) for *k* = *K*_*C*_ gives one more constraint, namely

$$\begin{array}{c} N_{C}=\frac{N_{S}r_{S}}{r_{C}}\cdot 2^{K_{C}-1}. \end{array}$$ (22)

These constraints can be used to set realistic values for the free parameters of the model. Importantly, the above steady state analysis is conditional on all *c*_*x*_ = 0 and hence ignores the feedback mechanism (Eq 14). The unconditional steady state is unfortunately more complicated to analyze, also because the feedback signal has a non-linear influence on the system. However, the hope is that setting parameters based on the conditional steady state analysis will still lead to useful values—which is indeed the case as shown next.

### Rough parameter estimates

The free parameters of the model are:

- the number of stem cells *N*_*S*_
- the target number of erythrocytes, granulocytes, and thrombocytes in the blood stream, *N*_*E*_, *N*_*G*_, and *N*_*T*_
- their death rates *r*_*E*_, *r*_*G*_, and *r*_*T*_
- the number *m*_*T*_ of platelets in each megakaryocyte
- the maximum proliferation rate of cells *r*_max_
- the sensitivity offset parameter *b*
- the increase in growth factor sensitivity over stages for each lineage *a*_*E*_, *a*_*G*_, and *a*_*T*_
- the number of stages for each lineage *K*_*E*_, *K*_*G*_, *K*_*T*_
- and for the common precursors *K*_*C*_
- and, finally, the feedback gain *δ*.

We will use clinical observations and current best estimates to set these free parameters. First, for the number of stem cell *N*_*S*_ we use the same estimate as Dingli et al. [13], namely that there are roughly 400 hematopoietic stem cells that are active at any one time [19]. For healthy adults

$$\begin{array}{ccc} N_{E} & \approx \;\text{5.0}\;\text{million}/\mu L\cdot 5L & \approx \;\text{25.0}\;\text{trillion}\;\text{erythrocytes}\;\\ N_{G} & \approx \;\text{4000}/\mu L\cdot 5L & \approx \;\text{20.0}\;\text{billion}\;\text{granulocytes}\;\\ N_{T} & \approx \;\text{250000}/\mu L\cdot 5L & \approx \;\text{1.2}\;\text{trillion}\;\text{thrombocytes}\; \end{array}$$

are realistic numbers that can be obtained from clinical norms and assuming 5 liters of blood in the body. Erythrocytes are estimated to die after 110 days on average (1/*r*_*E*_). For granulocytes the available estimates are highly variable. The latest estimates using in vivo labeling are 5 days, considerably higher than previous ex-vivo estimates [20]. However, these measurements were just for one type of granulocyte and other estimates suggest survival times of only 8 hours. We therefore decided to use a value in between, namely 2 days (1/*r*_*G*_). Classic data on thrombocytokinetics estimate that thrombocytes leave the blood stream after 10 days (1/*r*_*T*_). One complication with thrombocytes is that the last committed precursor in the bone marrow, the megakaryocytes, produce about a thousand platelets. Another complication is that megakaryocytes undergo endomitosis, i.e. each cell cycle doubles the chromosomes and nuclear units within the cell but there is no cell division. In the model we will not distinguish between normal mitosis and endomitosis and hence *m*_*T*_ is the number of thrombocytes in each nuclear unit of a megakaryocyte. This number is estimated to be around 50 [21]. In contrast, *m*_*E*_ = *m*_*G*_ = 1 as the last erythrocyte and granulocyte precursors divide normally. Finally, the cell cycle takes a certain time and therefore cells cannot divide at arbitrary rates. A realistic number for the maximum number of cell divisions per day, *r*_max_, is 5 [13].

The remaining parameters are more difficult to set to reasonable values as their relationship to more easily observable quantities can be non-linear and parameters interact, too. For example, from Eq (9) it follows that the offset parameter *b* and the slopes *a*_*x*_ are related to the log odds of the binding probability at each stage *j*, i.e.

$$\begin{array}{c} a_{x}j+b+c_{x}=\text{log}(\frac{p_{x,j}\left(c\right)}{1-p_{x,j}\left(c\right)}) \end{array}$$ (23)

for *x* ∈ {*E*, *G*, *T*}. For *j* = 0 we can, however, solve this equation for *b*. If all *c*_*x*_ = 0, the three probabilities in the stem cell compartment, *j* = 0, are equal, i.e. *p*_stem_ = *p*_*E*,0_(0) = *p*_*G*,0_(0) = *p*_*T*,0_(0). This is the situation when erythrocytes, granulocytes, and thrombocytes are all at their target values (Eq 14). With this assumption

$$\begin{array}{c} b=\text{log}(\frac{p_{\text{stem}}}{1-p_{\text{stem}}}). \end{array}$$

and using Eq 11 and again using the shorthand *r*_*S*_ = *r*_*C*,0_(0) we find that

$$\begin{array}{cc} r_{S} & =r_{\text{max}}\cdot (1-{\left(1-p_{\text{stem}}\right)}^{3})\\ {\left(1-p_{\text{stem}}\right)}^{3} & =(1-\frac{r_{S}}{r_{\text{max}}})\\ p_{\text{stem}} & =1-{\left(1-\frac{r_{S}}{r_{\text{max}}}\right)}^{\frac{1}{3}}. \end{array}$$ (24)

We already know *r*_max_ but what is the proliferation rate of the stem cells *r*_*S*_? The rate at which they divide is about once a year, i.e. 1/365. This is the same estimate as used by Dingli et al. [13] which is based on allometric scaling arguments [19]. It is in between other estimates that range from once every 25-50 weeks to 0.6 times per year [22, 23]. Anyway, with this number we can compute *p*_stem_ and we find that *b* ≈ −8.61.

With *b* known, what are the slopes *a*_*E*_, *a*_*G*_, and *a*_*T*_? We assume that we know the rate at the previous to last stage of each lineage (remember the last stage are the mature blood cells), the output rate into the blood stream: $r_{\text{blood}}=r_{E,K_{E}-1}=r_{G,K_{E}-1}=r_{T,K_{E}-1}=4$ times per day. This is chosen to be just below the maximum rate but leaving a little room to increase the rate for the feedback mechanism. With this assumption we immediately get from Eq (11) that $p_{\text{blood}}=p_{x,K_{C}+K_{x}-1}=r_{x,K_{x}-1}/r_{\text{max}}$. Which allows us to solve Eq (23) for *a*_*x*_:

$$\begin{array}{c} a_{x}=\frac{\text{log}(\frac{p_{\text{blood}}}{1-p_{\text{blood}}})-b}{K_{C}+K_{x}-1} \end{array}$$

for *x* ∈ {*E*, *G*, *T*}. Unfortunately we can only solve this if we know the number of stages *K*_*E*_, *K*_*G*_, *K*_*T*_, and *K*_*C*_. From Eq (21) we get that

$$\begin{array}{c} K_{x}=1-K_{C}+{\text{log}}_{2}(\frac{N_{x}r_{x}}{N_{S}r_{S}})-{\text{log}}_{2}\left(q_{x}\right)-{\text{log}}_{2}\left(m_{x}\right) \end{array}$$ (25)

for *x* ∈ {*E*, *G*, *T*}. Hence, we can only compute *K*_*x*_ if we know all *a*_*x*_ and *K*_*C*_ since the *q*_*x*_ depend on those. But remember that *a*_*x*_ in turn depends on all *K*_*x*_, including *K*_*C*_. Assuming that at least *K*_*C*_ is known, say *K*_*C*_ ≈ 14, these mutual dependencies suggest an iterative scheme to update each set of equations in turn. This scheme converges extremely quickly. If applied we find that *a*_*E*_ ≈ 0.25, *a*_*G*_ ≈ 0.29, and *a*_*T*_ ≈ 0.31. More importantly, we get estimates for the number of stages in each lineage, namely

$$\begin{array}{ccc} \;K_{E}\approx 27\; & \;K_{G}\approx 22\; & \;K_{T}\approx 19\; \end{array}$$

that we rounded to the closest integer. Hence, in order to become an erythrocyte a stem cell has to go through *K*_*E*_ + *K*_*C*_ ≈ 41 cell divisions. This number is considerably higher than previous estimates [13] (in their Eq (1)—that is analogous to our Eq (25) with *q*_*x*_ = 1 and *m*_*x*_ = 1—they do not have *r*_*S*_).

Due to the rounding of *K*_*x*_ these estimates will not produce the exact desired expected outflow *N*_*x*_*r*_*x*_ for the hematopoietic system that was used to fit the parameters. As we can only have an integer number of stages, the target numbers of erythrocytes, granulocytes, and thrombocytes are constrained by the integer powers in Eq (21). In order to make the *N*_*x*_ fit the desired values we adjust the *r*_*x*_, i.e. the death rates of erythrocytes, granulocytes and thrombocytes according to Eq (21). The new adjusted estimates are

$$\begin{array}{ccc} \;1/r_{E}\approx 104.41\;\text{days}\; & \;1/r_{G}\approx 1.62\;\text{days}\; & \;1/r_{T}\approx 11.20\;\text{days}\; \end{array}$$

which are still realistic compared to the initial values of 110, 2, and 10 days.

These estimates depend on knowing *K*_*C*_ that we have seemingly arbitrarily set to 14. Based on mutations that affect several lineages it has been estimated that there are between 20 and 100 thousand CFU-GEMM cells [13], i.e. last common progenitors. Say we want to choose *K*_*C*_ such that the expected number of cells at the last common precursor stage is approximately 60000. We can vary *K*_*C*_, fit all other parameters given *K*_*C*_ and compute the resulting conditional expected number of last common precursors $N_{C}=E\left[N_{C,K_{C}}|c=0\right]$ using Eq (22). We can then choose *K*_*C*_ such that *N*_*C*_ ≈ 60000. Using this procedure results in *K*_*C*_ ≈ 14, the number we have used above. The expected behavior of the model with all the parameters set as just described is shown in Fig 2. Recently, it has been suggested that the textbook view of many stages of pluripotent common progenitors in hematopoiesis may not be correct and that cells commit very early to one of the three lineages [24]. If this was true *K*_*C*_ would have to be chosen much smaller, perhaps even set to zero. In the following we will, however, stick to the textbook view.

#### Feedback and stem cell transplants

Finally, we set the gain of the feedback mechanism *δ*. If the granulocyte count drops to zero, as it can for example happen after radiation- or chemo-therapy, the stem cells should definitely proliferate more than at their steady state rate *r*_*S*_, but probably also not at the same maximum rate *r*_max_ that cells towards the end of the system work at (see Fig 2, panel B). Let $r_{S}^{\prime }$ be the rate that results in the case when all *c*_*x*_ = *δ* (see Eq 14). There is a one-to-one correspondence between $r_{S}^{\prime }$ and *δ* that can be obtained from Eqs (23) and (24) when substituting $r_{S}^{\prime }$ for *r*_*S*_ and *δ* for *c*_*x*_:

$$\begin{array}{cc} \delta =\text{log}(\frac{p_{\text{stem}}^{\prime }}{1-p_{\text{stem}}^{\prime }})-b & p_{\text{stem}}^{\prime }=1-{\left(1-\frac{r_{S}^{\prime }}{r_{\text{max}}}\right)}^{\frac{1}{3}}. \end{array}$$

Setting $r_{S}^{\prime }$ is more intuitive than setting *δ* directly. As the amplification of the output of the stem cell compartment is so massive (Eq 17) even a small increase from *r*_*S*_ to $r_{S}^{\prime }$ can lead to a big change in the blood counts. Also, normal fluctuations around the steady state should not lead to big fluctuations of the proliferation rate of the stem cells. Still, even with these constraints it is challenging to set $r_{S}^{\prime }$ without detailed data on the dynamics of the system. We will set this parameter by simulating a stem cell transplant and making sure that the time course of the simulated parameters roughly matches clinical observations. In this simulation, and most simulations to be reported below, we have set Δ*t* = 1/*r*_max_/5. We have also simulated most results of this paper with a Δ*t* twenty times smaller and do not find significant deviations in the results. For the few simulations that simulated several years we set Δ*t* = 1/*r*_max_.

**Fig 3:**
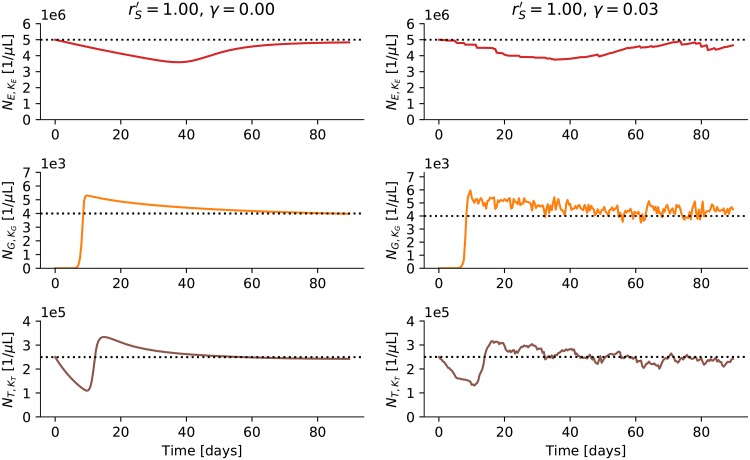
Simulation of stem cell transplant. The left side panels depict erythrocyte, granulocyte, and thrombocyte counts for a simulation of a stem cell transplant (for details see main text). The right side panels show the same with increased noise scaling.

The left side panels of Fig 3 depict the erythrocyte, granulocyte, and thrombocyte counts after a stem cell transplant that happened at *t* = 0. To simulate the stem cell transplant we first simulate the effect of chemo-therapy or radiation-therapy. After a couple of weeks of therapy we assume that all blood production came to a halt and the reserves in the bone marrow have been used up, i.e. there are no more cells at any of the stages of the model, except the blood stream. The erythrocytes and thrombocytes are assumed to be at normal levels because they have been transfused to stabilize the patient. Granulocytes, in contrast, cannot be transfused easily and hence because of their short half-life we assume they have been completely depleted (patients are treated with antibiotics to prevent infections). Then, at *t* = 0, a short time after the transplantation all stem cells are assumed to be back to their normal functioning and start replenishing the bone marrow. It then takes a while until the first fresh cells arrive in the blood stream. There is an overshoot in the granulocytes and thrombocytes and the system takes a while to return to the steady state. Erythrocytes respond a lot more slowly. The time until the system starts responding (the granulocyte counts start going up) shortens with an increase in $r_{S}^{\prime }$ but this also results in a bigger overshoot and a longer time to resettle. We have set $r_{S}^{\prime }=1.00$ by eye such that it takes less than two weeks until granulocytes and thrombocytes are back up but the overshoot does not become unrealistically large.

#### Comparison to clinical data

**Fig 4:**
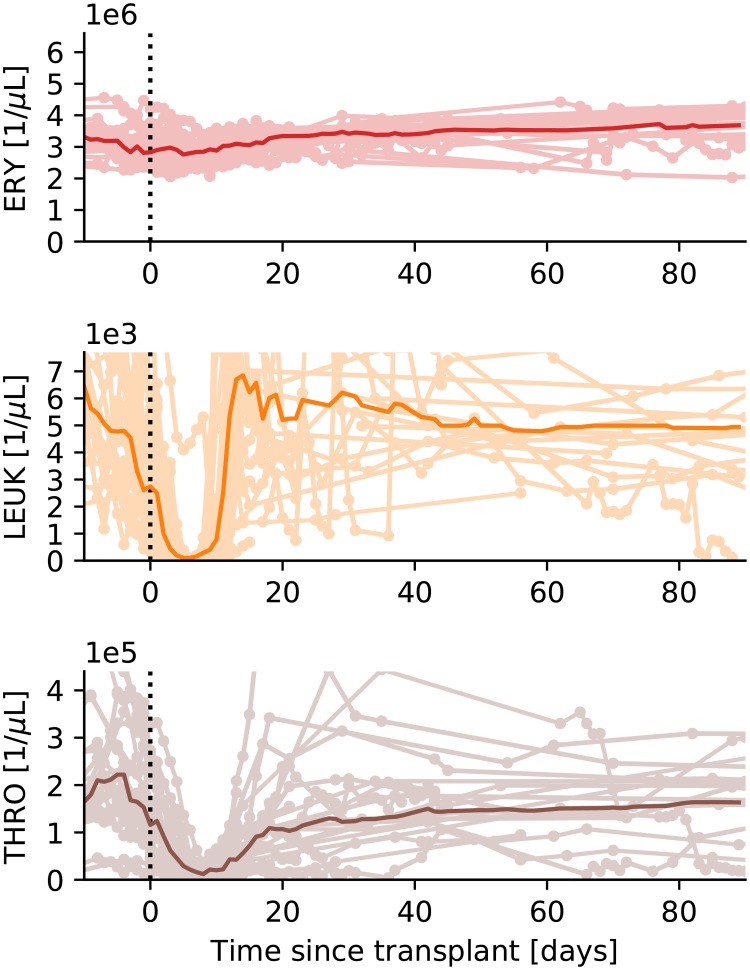
Clinical data from autologous stem cell transplants. The erythrocyte, leukocyte, and thrombocyte counts for 24 lymphoma patients around the time of their stem cell transplant are shown. The faint colors depict the individual patients with dots denoting actual measurements. The bold colors depict the median time-courses of linear interpolations of the single-patient data.

The time scale of less than two weeks is consistent with clinical experience simply because we have set the parameters accordingly. But do the details of the dynamics that our model shows fit clinical observations, too? This is not easy to answer. Allogeneic transplantation has the complication of graft versus host disease, hence it is simpler to consider autologous transplantation only. As a first validation of the model, we have therefore looked up clinical records of 24 lymphoma patients who received autologous hematopoietic stem cell transplantation after myeloablative conditioning. The time courses of their blood counts can be seen in Fig 4 along with the median time-course.

Consider the erythrocytes first. As expected, the model and the data show a very slow time-course with relatively small changes over the 90 days after the transplant. The main difference is that the patients are anemic. As we calibrated the model with data from healthy subjects it is not surprising that the absolute numbers are different.

As for the leukocytes: The simulation only models the granulocytes as the most frequent leukocytes and therefore the numbers in Figs 3 and 4 are not directly comparable but the median time-course of the overshoot and the slow resettling to the steady state are remarkably similar—even though we only gathered these data after having fixed the parameters.

The thrombocyte counts show a very different median time-course than expected. In particular, there is no overshoot in the clinical data. It could be that we simply need to change the gain parameter for the thrombocyte feedback loop separately from the other gains (Eq 14). However, in contrast to leukocytes, thrombocytes can be transfused and patients do receive transfusions during their treatment. Our simulation assumed therefore that patients go into the transplantation with a normal thrombocyte count. This is clearly not the case in the data. Patients receive transfusions at varying time-points during their treatments and these transfusions have a big effect on the thrombocyte counts. Also, patients vary in the time that passed between the myeloablative conditioning and the stem cell transplant. There might be an increased demand of thrombocytes during the transplant, too. Given all these uncontrolled factors, the median time-course of the patient data is not easily comparable to the model simulation for the thrombocyte counts. In general, given the large differences between single patients it seems desirable for future work to try to model single patients and not just prototypical behavior.

#### Noise scaling

A striking feature of the simulation depicted in the left panels of Fig 3 is that the curves are extremely smooth and the system seems to behave almost deterministically, despite the fact that random choices are made at every stage of the system. Apparently the numbers of cells are so big, the difference between inflow and outflow so small, and the feedback mechanism so effective that there is hardly any significant variation in cell counts. In fact, when running the simulation for a year in steady state the absolute average deviation from the target values is at most 0.026%. Such low variance in the blood counts is, of course, unrealistic. In reality there will be many more sources for variability in the data, e.g. additional noise in the growth factor concentrations due to infections or temporarily increased oxygen demand. The simulation should look more like the right panels in Fig 3. As the main source of variance in the model are the binomial random variables (Eq 2) one simple way to inject more noise into the system is to change the binomial to a beta-binomial to allow for overdispersion. This can be achieved by changing Eq (1) to

$$\begin{array}{cc} r_{x,k}^{\prime }\left(t\right) & ∼\text{Beta}(\frac{\alpha_{x,k}\left(t\right)}{\gamma },\frac{\beta_{x,k}\left(t\right)}{\gamma })\\ \alpha_{x,k}\left(t\right) & =r_{x,k}\left(c\left(t\right)\right)\\ \beta_{x,k}\left(t\right) & =1-\alpha_{x,k}\left(t\right) \end{array}$$

and in this way the expectation of *M*_*x*,*k*_(*t*) stays the same (Eq 2), hence keeping the conditional steady state analysis intact. The variance, however, increases with the noise scaling parameter *γ*. For *γ* → 0 the original model is recovered. In the limit of Δ*t* → 0 a beta process is obtained instead of the Poisson process of the original model [25].

**Fig 5:**
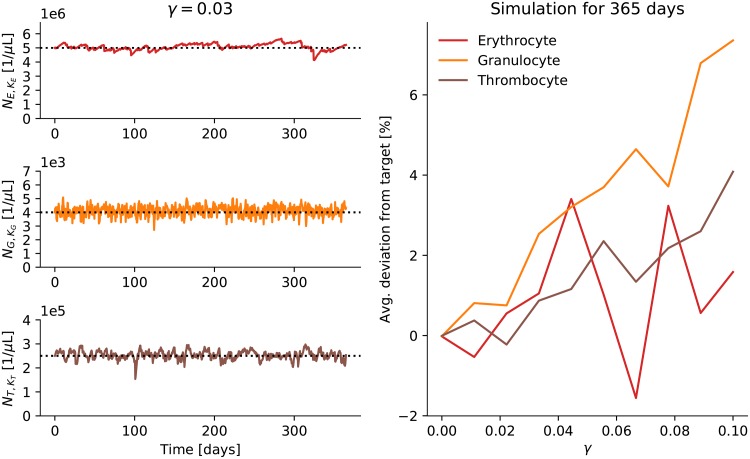
Deviation from target values. The panels on the left show the erythrocyte ($N_{E,K_{E}}$), granulocyte ($N_{G,K_{G}}$), and thrombocyte ($N_{T,K_{T}}$) counts for the hematopoietic system in steady state. The noise scaling parameter *γ* is varied in the panel on the right and the resulting average deviation of the counts from their target values is shown for one year-long run of the simulation.

Unfortunately, the introduction of the noise scaling parameter *γ* leads to shifts in the unconditional steady-state values of the erythrocyte, granulocyte, and thrombocyte counts away from their target values *N*_*E*_, *N*_*G*_, and *N*_*T*_ (Eq 14). The left side panels in Fig 5 show the steady state behavior of the system for *γ* = 0.03. There are small shifts of the average counts compared to the desired target values (the dashed horizontal lines) but they are hardly noticeable. In the panel on the right the relationship between *γ* and the expected values of the steady state behavior is explored systematically. The noise scaling parameter *γ* is varied and the deviation of the average counts from their target values over a simulation of one year is shown. The deviations increase with the noise scaling parameter. This effect can be explained by the non-linear feedback that quickly increases the proliferation rate of the stem cells whenever the output is too low but cannot decrease the proliferation rate to the same degree whenever the output is too high—simply because the proliferation rate of the stem cells is already very low for the target values. In the following we have set *γ* = 0.03. This value was chosen by eye to give a reasonable range for the variances of the erythrocytes, granulocytes, and thrombocytes but results only in a relatively small average deviation from the target values.

### Simulation of myeloid leukemia

Ultimately, models for hematopoiesis should be useful for understanding clinical conditions. As a first step in this direction, we will extend the model to capture some aspects of acute myeloid leukemia (cf. [16]). We assume leukemia develops through mutations in crucial pathways, the so-called hallmarks of cancer [8, 9, 26]. Here, we will focus on two such pathways: The pathway that controls the replicative capacity of a cell and the pathway that controls its further differentiation. We extend the above model of hematopoiesis by allowing for two kinds of mutations, one in each pathway.

**Fig 6:**
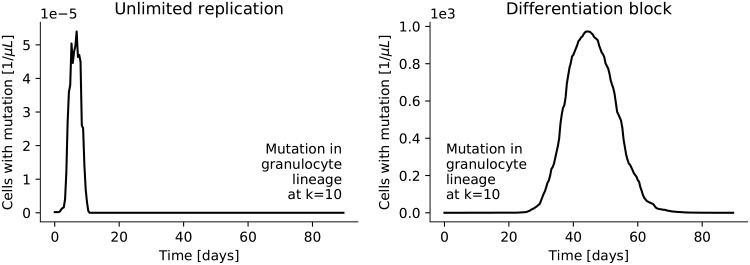
Single mutations. Mutations are introduced in the granulocyte lineage at stage *k* = 10 at time zero. In the left panel the mutation gives the mutated cell and all its daughter cells unlimited replicative capacity. The plot shows the number of cells with this mutation as a function of time (measured per microliter blood in the body). The clones quickly die out because they differentiate into mature granulocytes and are subsequently removed from the system. In the right panel the mutation blocks further differentiation but also these clones die out once they have exhausted their replicative capacity.

First, we allow for mutations that give cells unlimited replicative capacity. This requires the introduction of a limited replicative capacity for “normal” cells (which was ignored in [16]). Normal cells are limited in their replicative capacity by the Hayflick limit. We assume all cells that leave the stem cell compartment can replicate a fixed number of times, and this number is the same for all cells. This number has to be greater than the number of stages that the erythrocytes have to go through—otherwise no mature erythrocytes will be produced—but decreases with increasing age and for adults should therefore be lower than the absolute Hayflick limit of about 70. Here we have arbitrarily set it to 60. In the model we keep track of the remaining number of divisions for a cell by adding subcompartments to each stage accordingly: A compartment collects all cells at a certain stage with a certain remaining replicative capacity. For normal cells the remaining replicative capacity decreases as they differentiate further and cells that have exhausted their replicative capacity vanish from the simulation. Unlimited replicative capacity can now be modeled by simply not decreasing the replicative capacity of a cell. That is, the two children of a cell move on to the next differentiation stage without a decrease in their replicative capacity and so do all their children and all their children, and so on, giving rise to an exponential growth of cells with this particular mutation. For the left panel of Fig 6 such a mutation was introduced at the tenth stage of the granulocyte lineage. The number of clones with this mutation is shown as a function of time for one run of the simulation. Even though these cells have unlimited replicative capacity they quickly die out as the daughter cells eventually differentiate further into mature granulocytes that do not divide any more and are quickly removed from the blood stream.

Second, we allow cells to develop a complete differentiation block, i.e. they do not differentiate further. Instead all their daughter cells (and their children and so on) remain at the same stage of the hematopoietic system. A differentiation block leads to an exponential increase in cells at the stage where the block occurs. The proliferation rate is given by the proliferation rate of cells at this stage (see panel B in Fig 2). Hence, a mutation at an earlier stage will lead to a slower increase in clones than a mutation at a later stage. However, the exponential increase will eventually slow down and will be followed by a decrease of clones once the clones have exhausted their replicative capacity. This time course is depicted in the right panel of Fig 6. The peak number of cells depends on the stage where the mutation occurs. Later stages have already used up more of their replicative capacity and therefore cannot proliferate as much. In order to get a number of mutated cells in the bone marrow that is comparable in magnitude to the number of normal cells in the bone marrow during steady state the mutation has to occur at a very early stage.

**Fig 7:**
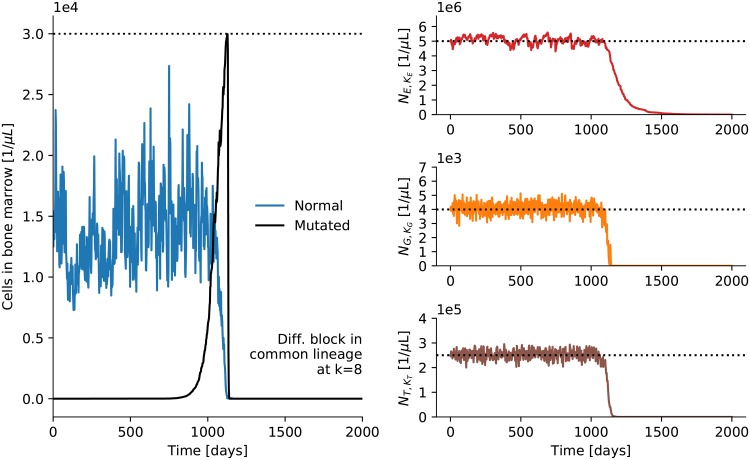
Early differentiation block. A differentiation block is introduced in the common precursor stage *k* = 8 at time zero. The blue plot in the left panel shows the number of normal cells in the bone marrow (per microliter blood in the body). After a nascent phase with slow growth the number of clones, shown in black, explodes. As in the right panel of Fig 6 the number of clones here is bound by a limited replicative capacity and comes down again. However, here we assume in addition that there is a limit to the number of cells that can be hosted by the bone marrow (dashed horizontal line) and excess cells are pushed into the blood stream. The panels on the right show the corresponding development of erythrocyte, granulocyte, and thrombocyte counts in the blood. After the bone marrow has been taken over completely by the mutated cells there is no more production of mature blood cells and hematopoiesis breaks down completely.

Say, a differentiation block occurs at the common precursor stage *k* = 8, much earlier than in Fig 6. As cells at this early stage have not used up as much of their replicative capacity as later cells they can produce a much larger number of descendant cells. Fig 7 shows that a differentiation block that occurs early can produce a number of clones that produce problems for hematopoiesis. In order to make the model a bit more realistic, we have assumed that there is a hard limit to the number of cells that can be hosted by the bone marrow, either because of space limitations or other finite resources. Excess cells—mutated and normal cells—are assumed to be pushed out of the bone marrow and into the blood stream at random. As one symptom of leukemia is the presence of immature blood cells in the blood stream this assumption seems reasonable. The pushed-out cells are subsequently removed from the blood stream just like other cells that do not belong there. For the simulation shown in Fig 7 we have set the limit for the number of cells in the bone marrow to 30000 per microliter, roughly twice the average number of cells in the bone marrow. This number was chosen to ensure that normal steady state fluctuations in the number of cells in the bone marrow do rarely lead to cells being pushed out. The left panel of Fig 7 shows the number of normal and the number of mutated cells in the bone marrow over time. The right panels of Fig 7 show the corresponding erythrocyte, granulocyte, and thrombocyte counts in the blood. As the mutated clones completely take over the bone marrow no more normal mature blood cells and platelets can be produced and hematopoiesis breaks down completely. The break-down is very sudden due to the exponential growth of mutated cells—consistent with the sudden onset of acute leukemia. Hence, in our simulations a *single* mutation that leads to a complete differentiation stop at an early stage can lead to acute leukemia.

**Fig 8:**
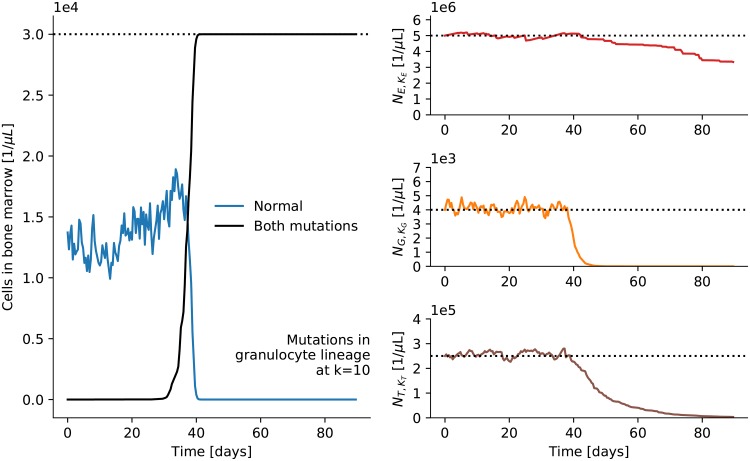
Late mutations. Mutations for unlimited replication and a differentiation block are introduced in the granulocyte lineage at stage *k* = 10 at time zero. The break-down of hematopoiesis happens much earlier than in Fig 7, where the mutation occurred earlier in the system.

In order to develop acute myeloid leukemia at a later stage of the hematopoietic system a cell has to acquire more than one mutation: It has to have unlimited replicative capacity and a differentiation block. Say both mutations come together in the granulocyte lineage at stage *k* = 10 and at time *t* = 0, where one mutation alone washes out quickly as shown in Fig 6). The left panel of Fig 8 shows how, after a much shorter nascent phase, the number of clones with both mutations grows extremely quickly until hematopoiesis breaks down (see right panels). Hence, if mutations for a differentiation block and unlimited replicative capacity come together, acute leukemia can also develop at later stages in our model. Still, importantly, all that is needed are two specific mutations in one cell.

The duration of the nascent phase and the growth rate of the mutated cells depend on the proliferation rate of cells at the stage where the differentiation block occurs. Hence, faster and slower growth correspond to differentiation blocks at later and earlier stages, respectively. This can also be seen when comparing the time axes of Figs 7 and 8. If limited replicative capacity is ignored, it is easy to derive an analytic expression for the expected time to diagnosis and the expected time until the complete break-down happens as a function of the stage where the mutations occur. We know the rate at which cells at that stage proliferate when the system is in steady state: *r*_*x*,*k*_ (see panel B Fig 2). The number of cells that are added in each time step follows Eq (2) and for sufficiently small Δ*t* the expected growth of mutated cells at this stage is therefore

$$\begin{array}{c} \underset{\Delta t\to 0}{\text{lim}}{\left(1+r_{x,k}\Delta t\right)}^{\frac{t}{\Delta t}}=e^{r_{x,k}t}. \end{array}$$

**Fig 9:**
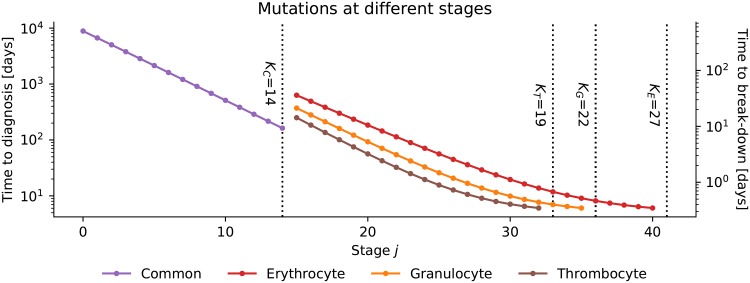
Time course of leukemia. Mutations for unlimited replication and a differentiation block are introduced at stage *j* at time zero. The time until leukemia is diagnosed is set to the time point when there are half as many mutated cells expected in the bone marrow as normal cells in steady state. This time is shown on the left y-axis. On the right y-axis the time from the diagnosis to the expected break-down of the bone-marrow is shown. This time point is assumed to be reached when there are twice as many mutated cells in the bone marrow as normal cells in steady state.

For a given number of mutated cells this equation can be solved for *t*, the time point at which this number is reached. We assume that the diagnosis is made when there are half as many mutated cells in the bone marrow as normal cells in steady state. The time until the diagnosis is made as a function of the stage where the mutations happen is shown on the left axis of Fig 9. Furthermore, we assume that the break-down happens when twice as many mutated cells occupy the bone marrow as normal cells in steady state. The time that passes between diagnosis and break-down is shown on the right axis of Fig 9. If at time *t* = 0 a cell with differentiation block and unlimited replicative capacity appeared at common precursor stage *k* = 1 (i.e. *j* = 1), it takes almost 20 years until the diagnosis is made. From this time point it takes about one year until the mutated cells have completely taken over the bone marrow.

One could have hoped that the only difference between acute and chronic leukemia is that chronic leukemia develops much more slowly because the mutations occur very early in the hematopoietic system. However, even for mutations that happen at the first stage of the system, one year after the diagnosis there are already twice as many mutated cells in the bone marrow than normal cells in steady state. While this is a much slower break-down of hematopoiesis than in Fig 8 it is still too fast for some cases of chronic leukemia.

Therefore, modeling chronic myeloid leukemia might require including the self-renewal mechanism of the original model of Dingli et al. [13] that we have ignored so far (cf. [16, 27–29]). The self-renewal parameter of the original model balances self-renewal and differentiation: While in our model a cell without a differentiation block always produces two daughter cells that are more differentiated than their parent, in the original model there is always the possibility that the two daughter cells are of the same type as their parent. In fact, in the original model all cells at all stages have a non-zero probability to self-renew. Hence, in the original model a differentiation block can be modeled in a graded fashion by simply increasing the probability for self-renewal at the cost of differentiation. In this way, the probability for self-renewal allows for a more fine-grained control of the growth of leukemic cells and also allows for much slower growth rates than depicted in Fig 9. Unfortunately, including self-renewal complicates the model further and requires changes to the parameter estimates, too. Furthermore, the self-renewal capabilities of non-stem-cell precursor cells are not very well understood and the assumption of the original model that all progenitors have the same capability to self-renew is at least questionable (see e.g. [30]). More experimental and theoretical work will be required before we feel comfortable to include self-renewal in our model. Alternatively, if the number of stem cells was larger than assumed here (based on the allometric scaling arguments of [19]), the proliferation rate of the stem cells could in fact be lower, while the expected outflow of the stem cell compartment stays the same. A lower proliferation rate for the early stages could thus also make the model more consistent with the slow progression of chronic myeloid leukemia without using an additional mechanism. Another simple fix could be to assume that leukemic cells have a proliferation rate that is slower than for normal cells at the same stage.

## Discussion

We have presented a model for myeloid hematopoiesis. It is based on the model of Dingli et al. [13] but extends it to explicitly include the erythrocyte, granulocyte, and thrombocyte lineages of blood formation. Considering different lineages complicates the model considerably compared to the original model. In particular, feedback in our model requires that different lineage-specific growth factor concentrations are taken into account. We have provided a conditional steady state analysis and we have shown that rough parameter estimates can be obtained from common clinical observations and readily available estimates from the literature.

For future work it will be desirable to obtain more quantitative data, i.e. beyond the blood counts for stem cell transplants already discussed, and use more principled statistical methods for parameter estimation that also produce confidence intervals (as e.g. in [17]). Such a more quantitative model should then also include the lymphoid side of blood production so that it can account for the majority of cells in a blood count.

Another direction for developing the model further is to use it to model cyclic neutropenia. The original model of Dingli et al. [13] has already been extended to capture cyclic neutropenia by also including a feedback mechanism [15], but our feedback mechanism is harder to analyze due to its non-linearity. Nevertheless, with some effort their insights can probably be transferred to our model.

Here, we have concentrated on modeling acute myeloid leukemia and extensions that seemed necessary to understand it. One such extension was the addition of a limited replicative capacity for normal cells—a property that up to now was ignored by similar models of acute myeloid leukemia (e.g. [16]). This part of the model should be made more realistic by following the lead of Werner et al. [17] who measured and modeled the distribution of telomere lengths in blood cells. They find, as expected, that with age telomere length—and therefore replicative capacity—decreases and they also show in their model that this effect can be explained by telomere shortening in the stem cell compartment. In brief, they find that telomere length decreases by roughly 50-75 base-pairs per year, which under the assumption that one cell division takes off around 50 base-pairs means that all stem cells replicate about once a year (see also [31, 32]).

A back-of-the-envelope calculation shows that the estimates that we use are, however, inconsistent with these results. We assume that there are 400 stem cells and that their proliferation rate is once a year [13, 19]. Hence, according to current estimates [17] roughly all of the 400 cells reduce their replicative potential by 1 each year. If we only had these 400 stem cells then after 30 years we could not produce any more erythrocytes as the absolute Hayflick limit is around 70 and there are about 40 stages from the stem cell compartment to the erythrocytes in our model. Luckily, the current best estimates for the overall number of hematopoietic stem cells is around 12000—of which only 400 are active at any one time (see also [19]). If we assume that the 400 active stem cells take turns, i.e. a cell that replicated is replaced by a fresh cell from the full pool, then it will take 30 years until each of the 12000 cells has divided itself once. Hence, even after 90 years the stem cells will only have used up 3 of their 70 cell divisions given by the Hayflick limit. This is, however, inconsistent with the observed telomere shortening of around 50 base-pairs per year in mature blood cells, because this shortening implies one cell division of all cells in the full stem cell pool per year. So perhaps 400 active stem cells is too small a number and the allometric estimates on which this number is based cannot be trusted. It is thus crucial as one of the next steps to combine models for telomere shortening [17] with models of hematopoiesis and jointly estimate the number of active stem cells, their proliferation rates and the number of stages from quantitative data that include telomere distributions and blood counts. Importantly, the data that go into these models and the resulting latent parameter estimates have to be accompanied by uncertainty estimates. Otherwise it is impossible to tell whether the described inconsistencies are real or whether they are well within plausible ranges given the available data. For stochastic models like Dingli’s or ours it is thus very desirable to go beyond quick-and-dirty parameter estimates and use fully Bayesian inference methods in the future (as in [17]).

Finally, as we assume in our model that leukemia is the result of mutations in specific pathways that control a cell’s replicative capacity and its differentiation—in line with the conception of cancer hallmarks [8, 9, 26]—it would be desirable to link the model to genetic data. It has been suggested that only two to eight driver mutations typically lead to tumorigenesis and that only 12 signaling pathways with about 140 genes might be involved [10]. For acute myeloid leukemia these genes and mutations have recently been catalogued systematically [7] and it might hence be possible to get quantitative estimates for the probability of, for example, an acquired differentiation block.

Even without precise numbers one can make some qualitative inferences: Just like the model of Dingli et al. [13] our model suggests that many more mutations should occur at the later stages of the hematopoietic system than at the early stages, simply because there are many more cell divisions at the later stages. In fact, at about stage *j* = 25 the expected outflow is 10^7^ cells per day (see panel C in Fig 2) and all these cells divide. Assuming that 10^−7^ mutations happen per gene per cell division [33] we thus expect one mutation per gene per day just for cells at this stage (cf. [34] where only stem cell divisions are considered). Luckily, mutations in just one gene at a late stage will quickly wash out as we have shown above for unlimited replicative capacity and differentiation block (see Fig 6). And mutations at an earlier stage, where the mutation has a bigger impact (see Fig 7), are less likely due to the lower number of cell division at earlier stages. Still, given that mutations should be extremely common in the hematopoietic system it seems likely that combinations of several mutations, like a differentiation block together with unlimited replicative capacity, can still occur relatively frequently at later stages. The exact probability to encounter combinations of mutations is hard to compute though as the order of acquiring the mutations matters. For example, on the one hand, as an unlimited replicative capacity washes out more quickly than a differentiation block (Fig 6) it seems more likely that the differentiation block was acquired first. On the other hand, an unlimited replicative capacity at an early stage of the system produces a huge number of descendants that can also acquire a differentiation block. Understanding such dependencies between mutations might ultimately guide the design of therapeutic protocols that target the effects of mutations in different pathways in different orders.
